# When Forgetting Preserves Memory

**DOI:** 10.3389/fpsyg.2013.00032

**Published:** 2013-02-04

**Authors:** Almut Hupbach

**Affiliations:** ^1^Department of Psychology, Lehigh UniversityBethlehem, PA, USA

**Keywords:** memory reconsolidation, intentional forgetting, memory modification, list learning, episodic memory

## Abstract

There has been a resurgence of interest in defining the circumstances leading to memory modifications. Studies have shown that reactivating a supposedly stable memory re-introduces a time-limited window of plasticity during which presentation of interfering material can cause long-term memory changes. The present study asks whether such memory changes can be prevented if people are instructed to forget the memory before the new material is encoded. Participants learned a set of objects. After 48 h, they were reminded of this learning episode, and learned another set of objects. Again 48 h later, they recalled the first (Exp. 1) or second set (Exp. 3). As shown previously, a reminder caused intrusions from the second set into recall of the first set. Here I show that the instruction to forget the first set significantly diminished intrusions from the second set, especially when the instruction was given before the new set was encoded in the second session. Experiment 2 suggests that the reduced intrusions were due to list segregation/isolation, rather than temporarily inhibited access to Set 1. Taken together, the study shows that the attempt to forget a memory can immunize it such that the presentation of interfering material has limited effects, and the memory can be recalled unchanged in the future. This is important when veridical memory is essential, such as in eyewitness testimonies.

## Introduction

Memory does not always provide an accurate record of the past. For instance, misinforming people about details of a witnessed event can cause them to adopt these false details as their true memories (*the misinformation effect*, see e.g., Loftus, 2005 for a review). Memory reconsolidation might account for such memory changes (Hardt et al., 2010). The reconsolidation account proposes that every time a memory is reactivated (e.g., by questions about a previous event), the memory becomes fragile again and can be modified or updated. In order for the memory to “survive,” it needs to undergo a re-stabilization process called reconsolidation. This process has been extensively studied in animals (for reviews, see e.g., Nader and Hardt, 2009; Besnard et al., 2012), illuminating its behavioral consequences as well as the molecular and cellular underpinnings. Much less is known about reconsolidation in humans (cf. Schiller and Phelps, 2011). Hupbach et al. (2007) developed a paradigm to study reconsolidation-type processes in human episodic memory: participants learn a set of objects in Session 1. Forty-eight hours later (Session 2), they are either reminded of the previous learning episode or not, and then learn a second set of objects. When asked to recall Set 1 in Session 3 another 48 h later, reminded participants show a high number of intrusions from Set 2 into Set 1, while participants who were not reminded show very few intrusions. Importantly, this effect happens involuntarily and participants are not aware of intrusions. In fact, they are highly confident that intruded Set 2 items belong to Set 1 (Hupbach et al., 2009). We have interpreted the intrusion effect as evidence for memory reconsolidation: when Set 1 is reactivated during Session 2, memory for Set 1 re-enters a vulnerable, plastic state in which new items can be incorporated into the reactivated memory representations.

In contrast to the involuntary, automatic modification of memory that is common to the effect described above, memories are also under voluntary control, at least to some extent, as shown by research on retrieval suppression (for a review, see Anderson and Levy, 2009), and directed forgetting (DF, for reviews see, e.g., MacLeod, 1998; Bäuml, 2008). In the present study I ask whether involuntary memory changes can be prevented, and whether memories can be preserved in its original form, when people are instructed to forget the original memory before potentially interfering material is presented. More specifically, I combined the reconsolidation paradigm (that leads to integration of new information into the reactivated memory) with a list-wise DF paradigm (in which people actively attempt to forget a recently encoded memory).

In the so-called list-method DF paradigm, participants are either instructed to forget a just learned list because it will supposedly not be tested later, or to continue to remember this list for a later memory test. Then, all participants are asked to memorize a second list. In comparison to the remember condition, the instruction to forget causes impaired memory for the first list (costs), and improved memory for the second list (benefits of directed forgetting). Initially, it was assumed that the forget cue causes List 1 to be differentiated or segregated from List 2 items, which decreases proactive interference and allows participants to devote all their mnemonic activities to selectively rehearse List 2 (Bjork, 1970). Retrieval inhibition was later introduced as the crucial mechanism, and has remained the most widely accepted explanation for the DF effect (Geiselman et al., 1983; see also Bäuml, 2008). According to the retrieval inhibition account, the forget cue temporarily inhibits or blocks retrieval routes to the to-be-forgotten information, while leaving the actual strength of the memory unaffected.

Building on the original list segregation theory by Bjork (1970), Sahakyan and Kelley (2002) proposed the contextual change hypothesis, an alternative account of list-wise DF. They assume that the forget instruction causes an internal context shift, which participants accomplish by either focusing on something task-unrelated or deliberately sampling new contextual elements. Set differentiation is assumed to be a function of the context shift. Set differentiation is, however, not sufficient for the DF effect. Critically, the context at retrieval mismatches the List 1 encoding context, which negatively affects recall of List 1. Support for this account comes from the finding that when participants are instructed to mentally reinstate the List 1 encoding context, memory improves (Sahakyan and Kelley, 2002). Furthermore, interventions that potentially change the internal context have similar effects as the forget cue, such as imagining being invisible, mentally walking through your parents’ house (Sahakyan and Kelley, 2002), daydreaming (Delaney et al., 2010), chatting with the experimenter or wiping a computer screen (Mulji and Bodner, 2010).

Taken together, research shows that memories are often involuntarily modified (and unnoticed by the beholder), but that modifications can also be intentionally initiated. This raises the interesting question whether involuntary changes could be prevented if people engaged in intentional processes counteracting those changes. Reconsolidation research has shown that reactivated memories are fragile and prone to modification. Maybe modification of those memories can be prevented if people are asked to suppress or forget those reactivated memories. The present study tests this hypothesis.

## Experiment 1

In most list-wise DF studies, the forget cue follows List 1 encoding, and immediately afterward, List 2 is presented. After a brief interval, participants are then asked to recall the first or the second list. In addition to the difference in material (objects instead of words that are commonly used in DF studies), the object-learning paradigm differs in two other important ways from those studies: (1) final recall of Set 1 is not tested immediately after Set 2 presentation, but is tested in a third session that takes place 48 h after Session 2, (2) the two sets of objects are not presented in a single-session, but in two separate sessions with a 48 h delay separating them. Therefore, it was less clear when exactly to present the forget cue in the time interval between Set 1 and Set 2 learning. I decided to instruct participants to forget Set 1 either in Session 1, immediately after they had learned Set 1, or in Session 2, before they encoded the second set of objects. Presenting the forget cue after Set 1 encoding could affect post-learning stabilization processes for Set 1 memory (i.e., memory consolidation; see e.g., review by McGaugh, 2000). Instructing participants to forget Set 1 in Session 2, before presenting the second set, could inhibit or block access to Set 1 memory (e.g., Geiselman et al., 1983). This in turn could prevent its modification, such that the usual intrusion effect would not be observed. Both possibilities were tested in Experiment 1.

In all experiments in the present study, I used a spatial context reminder to reactivate memory for Set 1 in Session 2. This reminder involves bringing participants back to the same room (spatial context) in which they learned Set 1. This reminder has been proven to be the crucial component necessary to reactivate Set 1 memory and to cause intrusions from Set 2 into Set 1 (Hupbach et al., 2008). Additionally, participants worked with the same experimenter on all 3 days.

### Experiment 1A

#### Methods

##### Design and participants

All participants took part in three separate sessions. The temporal placement of the forget cue was the only independent variable that was varied between-subjects. I presented participants with the instruction to forget Set 1 either after they learned the first set of objects in Session 1 (FC-1), or before they learned the second set of objects in Session 2 (FC-2), or not at all (control). The last group’s performance provided a control against which I compared the experimental groups’ memory performance. Thirty-six undergraduate students (age range 18–35; 20 females, 16 males) from Lehigh University participated in the experiment. They received course credit or financial compensation for participation. Twelve participants were randomly assigned to each condition. All experiments were approved by the Institutional Review Board, and participants gave written consent before the experiment started.

#### Materials

Set 1 and Set 2 materials each consisted of 20 unrelated objects. Set 1: balloon, bow, calculator, toy car, crayon, cup, dice, feather, flashlight, flower, glue, key, sock, sponge, spoon, sunglasses, teabag, tennis ball, toothbrush, whistle. Set 2: apple, band-aid, battery, book, cassette tape, cellular phone, comb, dollar bill, elephant, envelope, paper clip, puzzle piece, rock, shovel, straw, thread, tissue, toy pot, watch, zipper.

#### Procedure

The experimental conditions for all experiments are outlined in Table 1.

**Table 1:** **Overview of experimental conditions in experiment 1a, 1b, 2, and 3**.

Experiment	Session 1	Session 2	Session 3
Exp. 1a 3 groups: FC-1, FC-2, control	*Encode set 1* FC-1: “I would like you to forget the objects you just learned. On Wednesday you will learn a new set of objects, and attempting to remember today’s set may interfere with your memory for the 2nd set on Wednesday. Today’s set is not important to remember for the rest of the experiment, but the set you will learn on Wednesday will be important to remember.”	FC-2: “I would like you to forget the objects you learned on Monday. Today you will learn a new set of objects, and attempting to remember the objects from Monday may interfere with your ability to memorize today’s objects. The objects from Monday are not important for the rest of the experiment, but the objects you learn today will be important to remember” Control: No special instruction *Encode Set 2*	*Recall set 1*
Exp. 1b 3 groups: FC-1, FC-2, R	*Encode set 1* FC-1: see above	FC-2: see above R: “The objects you learned on Monday are still important to remember for a later memory test. Today, I will present a second set of objects to you that you should also keep in memory. In other words, both sets of objects are important to remember.” *Encode set 2*	*Recall set 1*
Exp. 2 2 groups: FC-2, R	*Encode set 1*	FC-2: see above R: see above *Encode Set 2* *Recall Set 1*	
Exp. 3 3 groups: FC-1, FC-2, control	*Encode set 1* FC-1: see above	FC-2: see above R: see above *Encode set 2*	*Recall set 2*

The three sessions took place on Monday, Wednesday, and Friday of the same week, i.e., sessions were separated by 48 h. All sessions were administered in the same room by the same experimenter. At the beginning of Session 1, participants were told that they would have to memorize different lists of objects in each session. Participants were tested one at a time.

*In Session 1*, the experimenter pulled out one object at a time from a bag in random order and placed it in a distinctive basket. Participants were asked to name each object and remember it for a later memory test. After all 20 objects (Set 1) were placed into the basket the experimenter hid the basket and asked the participants to remember as many objects as possible. This procedure was repeated until the participants remembered at least 17 of the 20 objects or until a maximum of four learning trials was reached. Afterward, participants in the FC-1 group were told to forget the objects they had just learned, because memory for these objects would not be tested again, and because forgetting them would optimize learning of the new set in the next session.

Forty-eight hours after Session 1, *Session 2* was administered, in the same room and with the same experimenter as before. Thus, all participants received a spatial context reminder (Hupbach et al., 2008). Participants in the FC-2 group were told to forget the objects from Session 1, because memory for these objects would not be tested again, and because it would help them learn the new set. The control group did not receive any specific instructions concerning Set 1. All participants then encoded a second set of 20 objects (Set 2). All objects were placed in front of the participants, who were asked to name each object. After all objects had been named, participants were given another 30 s to study them. Then, the experimenter removed the objects, and asked the participants to recall as many of the objects as possible. The study procedure was repeated until participants recalled at least 17 objects, or for a maximum of four learning trials.

*In Session 3*, participants were instructed to recall as many objects as possible from Session 1 only, and the experimenter noted the remembered objects. This procedure was repeated for a total of four consecutive recall trials, with 30-s delays between trials.

#### Results

##### Learning set 1 and set 2

In order to assess potential differences in initial learning between the different groups, I analyzed the number of trials to reach criterion (criterion: recall a minimum of 17 objects). A 3 (Condition: FC-1, FC-2, control) × 2 (Set: Set 1 vs. Set 2) mixed ANOVA revealed a significant main effect of Set [*F*(1, 33) = 4.83, MSE = 0.414, *p* < 0.035]. Participants needed fewer encoding trials to learn Set 2 (*M* = 2.56) than to learn Set 1 (*M* = 2.89), reflecting either a general training effect, and/or that the learning procedure for Set 2 was superior to the learning procedure used for Set 1 encoding. Importantly, learning was comparable in all three conditions: there was no main effect of Condition, and no interaction between Set and Condition (both *F*s < 1).

##### Set 1 recall

**Figure 1:**
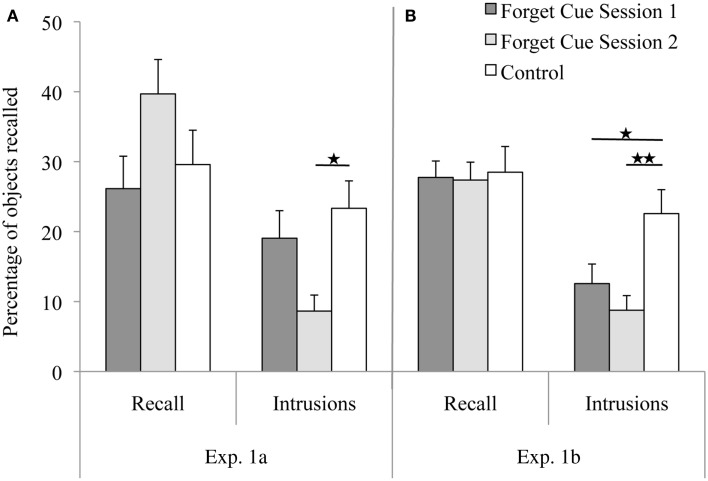
**Mean percentages of objects correctly and falsely recalled in the different experimental groups in (A,B): the forget cue was either given at the end of Session 1 or at the beginning of Session 2**. In **(A)**, the control group did not receive any instructions about Set 1, whereas in **(B)**, the control group was instructed to keep remembering Set 1 at the beginning of Session 2. Error bars represent standard errors of means. *Note:* Participants were asked to recall objects from *Set 1*. Objects that were falsely recalled from Set 2 are labeled as Intrusions.

The mean percentage of objects recalled from Set 1 and the mean percentage of objects falsely recalled from Set 2 (intrusions) are displayed in Figure 1A. The number of objects recalled from Set 1 were analyzed with a mixed ANOVA with Condition (FC-1, FC-2, control) as the between-subjects variable and Retrieval Trial (1–4) as the within-subjects variable. There was a significant effect of Trial [*F*(3, 99) = 9.74, MSE = 1.23, *p* < 0.01], reflecting a linear increase in recalled items with trials [*F*(1, 33) = 18.75, MSE = 1.80, *p* < 0.01]. Neither the main effect for Condition nor the interaction between Trial and Condition reached significance (*F* ≤ 2.31, *p* ≥ 0.12).

##### Intrusions

The number of intrusions from Set 2 into Set 1 recall were also analyzed with a mixed ANOVA. There was a significant effect of Trial [*F*(3, 99) = 3.29, MSE = 1.63, *p* = 0.024], reflecting a linear increase in recalled intrusions with trials [*F*(1, 33) = 4.40, MSE = 2.79, *p* = 0.044], but no Trial by Condition interaction (*F* = 1.13, *p* = 0.35). Most importantly, there was a significant effect of Condition [*F*(1, 33) = 4.75, MSE = 23.09, *p* = 0.015]. *Post hoc* comparisons (Tukey) revealed that the FC-2 group had significantly less intrusions than the control group (*p* = 0.014), and marginally less intrusions than the FC-1 group (*p* = 0.10). The FC-1 and control group did not differ in their rate of intrusions (*p* = 0.66).

#### Discussion

The control group replicated previous findings that reminders (spatial context & experimenter) cause new information to be incorporated into the reactivated memory (e.g., Hupbach et al., 2007): I found significant intrusions from Set 2 into Set 1 recall. The forget cue did not affect the amount of objects correctly recalled from Set 1, regardless of when it was presented (numerically, the group that was instructed to forget Set 1 in Session 2 recalled more objects than any other group, but this difference was not statistically significant and was not replicated in Exp. 1b).

Instructing participants to forget Set 1 in Session 1 did not affect the amount of intrusions (but see Exp. 1b). Importantly, however, instructing people to forget Set 1 prior to Set 2 encoding in Session 2, i.e., 48 h after Set 1 was learned, caused intrusions to drop significantly. This suggests that the forget cue can prevent memory modification when administered right before learning new information. Because this is a new finding, and the sample size of Experiment 1a was rather low, I decided to carry out a replication of the experiment with an increased sample size and a slightly different control condition. In DF studies, the forget instruction is usually compared to a remember instruction in which people are told to keep remembering List 1 while learning List 2. Accordingly, the control group in Experiment 1b was told at the beginning of Session 2 (before encoding Set 2) that the previously learned Set 1 was still important to keep in memory. The remember instruction was not given at the end of Session 1, because a pilot study had shown that this caused people to rehearse Set 1 between Session 1 and Session 2, i.e., outside of experimental control.

### Experiment 1B

#### Methods

The same method was used as in Experiment 1a, with the only exception that the control group was instructed to keep remembering Set 1 before learning Set 2 in Session 2. Thus, the only independent variable I varied between-subjects was the temporal placement of the forget cue, which was either given after learning the first set of objects in Session 1 (FC-1), or before learning the second set of objects in Session 2 (FC-2), or participants were instructed to keep remembering Set 1 at the beginning of Session 2 (R-group).

Sixty undergraduate students (age range 18–35; 27 females, 33 males) from Lehigh University participated in the experiment. They received course credit or financial compensation for participation. Twenty participants were randomly assigned to each condition. One participant (in the remember group) was excluded from the final data set, because he stated that he had rehearsed the objects between sessions. Thus, there were 20 participants in each the FC-1 and FC-2 group, and 19 participants in the R-group.

#### Results

##### Learning set 1 and set 2

In order to assess potential differences in initial learning, I analyzed the number of trials to reach criterion (criterion: recall a minimum of 17 objects). A 3 (Condition: FC-1, FC-2, R) × (Set: Set 1 vs. Set 2) mixed ANOVA revealed a significant main effect of Set [*F*(1, 56) = 4.44, MSE = 0.774, *p* = 0.04]. Participants needed fewer learning trials to learn Set 2 (*M* = 3.37) than to learn Set 1 (*M* = 3.71), replicating Experiment 1a. Additionally, as in Experiment 1a, learning was comparable in the three groups: there was no main effect of Condition, and no interaction between Set and Condition (*F*s < 1).

##### Set 1 recall

The number of objects recalled from Set 1 (see Figure 1B) was analyzed with a mixed ANOVA with Condition as the between-subjects variable and Trial (1–4) as the within-subjects variable. As in Experiment 1a, there was a significant effect of Trial [*F*(3, 168) = 15.94, MSE = 1.55, *p* < 0.01], reflecting a linear increase in recalled items with trials [*F*(1, 56) = 36.93, MSE = 1.89, *p* < 0.01]. Neither the main effect for Condition nor the interaction between Trial and Condition reached significance (*F*s < 1).

##### Intrusions

The number of intrusions from Set 2 into Set 1 recall (see Figure 1B) was also analyzed with a mixed ANOVA. There was a significant effect of Condition [*F*(1, 56) = 6.35, MSE = 24.83, *p* < 0.01]. *Post hoc* comparisons (Tukey) revealed that both forget groups significantly differed from the remember group (*p* = 0.04 for FC-1, *p* < 0.01 for FC-2), but there was no difference between the two forget groups (*p* = 0.60). Neither the main effect of Trial nor the interaction between Trial and Condition reached significance (*F*s < 1).

#### Discussion

Experiment 1b confirmed that the forget instruction does not impair recall of Set 1, but significantly reduces the number of intrusions from Set 2 into Set 1. The control groups of Experiment 1a (no forget cue) and 1b (remember-cue) showed very similar results, which suggests that the remember instruction did neither influence the veridical recall of Set 1 nor did it alter intrusions.

##### Recall of set 1

The finding that the forget instruction did not affect recall of Set 1 when given immediately after Set 1 was encoded suggests that a forget cue *per se* does not affect post-encoding consolidation processes (see also Bjork and Geiselman, 1978). Pastötter and Bäuml (2007, 2010) have shown that forgetting critically depends on the presentation of new information after the forget cue, and that the amount of List 2 encoding determines the magnitude of List 1 forgetting. Experiment 1 expands these findings by showing that List 2 has to be encoded shortly after List 1 was learned. Future research needs to define the critical time window during which List 2 encoding needs to take place.

Additionally, in most directed forgetting studies final recall follows shortly after List 2 presentation. Hence, the internal context during retrieval matches List 2 but mismatches List 1 encoding context, which in turn leads to impaired recall for Set 1 (context change account, Sahakyan and Kelley, 2002). In the current study, recall was assessed 2 days after Set 2 was learned. The mental context during retrieval is therefore different from the encoding context in all of the groups, which explains why I did not find impaired recall for Set 1 in the forget groups in comparison to the control groups. The failure to observe such long-term costs of directed forgetting also fits with the retrieval inhibition account which assumes that the inhibition that is induced by the forget instruction and that is responsible for the impoverished recall of List 1 is only temporary in nature. Importantly, in the present study the spatial context during recall always matched the encoding context, which might also explain the lack of retrieval costs. I will return to the interdependence of spatial and mental context in the General Discussion.

##### Reduction of intrusions

Although the forget cue did not impair Set 1 recall, it significantly reduced the amount of intrusions from Set 2 into Set 1, i.e., it prevented memory modification. This was observed in both Experiment 1a and 1b when the forget cue was given in Session 2. When the forget cue was given immediately after encoding of Set 1, the results were mixed: in comparison to the remember group, I found diminished intrusions in Experiment 1b, but not in Experiment 1a. Taken together, the forget cue reliably reduces intrusions when given shortly before encoding of potentially interfering material, but less reliably when given immediately after encoding of the original memory set. In what follows, I will discuss the inconsistent findings of the FC-1 group, and possible explanations for the reduced intrusions in the FC-2 group.

The retrieval inhibition account offers an explanation for the low intrusion rates in the FC-2 group, which I consistently observed in Experiment 1a and b. Potentially, participants responded to the forget instruction by engaging in inhibitory processes which temporarily reduced access to Set 1 memory, thus “deactivating” rather than reactivating it. In turn, this inhibition or deactivation put Set 1 memory in a “safe” place, which prevented the incorporation of new items into the memory. If this explanation is correct, and the forget cue indeed blocked retrieval routes to Set 1, then recall of Set 1 should be temporarily impaired if I tested it immediately after Set 2 learning at the end of Session 2. This was done in Experiment 2. The retrieval inhibition, however, cannot explain why the forget cue also diminished intrusions when it was presented right after Set 1 learning (although less so and only in Exp. 1b) – inhibition should have been released by the time participants returned for the second session. It is possible, however, that some participants remembered the forget instruction from the previous session, and thus “reinstated” it before learning Set 2.

An alternative explanation for the reduced intrusions can be delineated from theories that assume that the forget cue causes set differentiation or segregation, such as Bjork’s (1970) original theory of DF or Sahakyan and Kelley’s (2002) contextual change account. While Bjork did not specify the mechanism, Sahakyan and Kelley (see also Lehman and Malmberg, 2009) propose contextual differentiation as the critical factor driving list segregation. The assumed mental context change that follows the forget cue segregates List 1 from List 2, which decreases proactive interference from List 1 and diminishes cross-list intrusions (especially when item-specific encoding processes are used, Sahakyan, 2010; Sahakyan and Delaney, 2010). As stated above, because most directed forget studies are carried out as single-session experiments, both components are reflected in the outcomes, i.e., reduced memory expression (costs) and reduced intrusion (although the latter less consistently, see Sahakyan and Delaney, 2010 for discussion). In contrast, when spread out over multiple sessions as in the object-learning paradigm, the costs of DF are less likely to be found, because the retrieval context (in this case the mental or internal context) has changed for both the remember and the forget group after a delay. Thus, the costs of DF might be short-lived, but the effects of the enhanced list segregation – which are reflected in the reduced intrusions from List 2 into List 1 – are likely to remain long-term.

How can one differentiate between the retrieval inhibition and list separation accounts as explanations for the reduced intrusions? As stated above, in order to explain the reduced intrusions, the retrieval inhibition account would predict that access to List 1 was inhibited in Session 2, which should be reflected in diminished recall of Set 1. In contrast, according to the contextual change account, access to Set 1 in Session 2 should not be impaired in comparison to a remember group, because presumably, the internal context has changed for both the remember and the forget group after a 48 h delay, and thus, does not match the encoding context for Set 1 in either group. Experiment 2 tested whether a forget instruction impairs Set 1 recall when participants are asked to recall Set 1 immediately after encoding Set 2 in Session 2.

## Experiment 2

### Methods

The same methods as in Experiment 1b were used, with the exception that participants were asked to recall of Set 1 in Session 2, immediately after they had learned Set 2. Two experimental groups were tested: One group received a forget cue, and one a remember-cue at the beginning of Session 2, i.e., before learning Set 2.

Twenty-eight undergraduate students (age range 18–35; 13 females, 15 males) from Lehigh University participated in the experiment. They received course credit or financial compensation for participation. Fourteen participants were randomly assigned to each condition.

### Results

#### Learning set 1 and set 2

In order to assess possible differences in initial encoding, the number of trials to reach criterion (criterion: recall a minimum of 17 objects) was analyzed. A 2 (Condition: forget, remember) × 2 (Set: Set 1 vs. Set 2) mixed ANOVA did not reveal any significant effects (*F*s ≤ 1.74, *p* ≥ 0.20). Thus, although the numerical difference was in line with Experiment 1, the difference was not significant (Set 2: *M* = 3.43, Set 1: *M* = 3.75).

#### Set 1 recall

**Figure 2:**
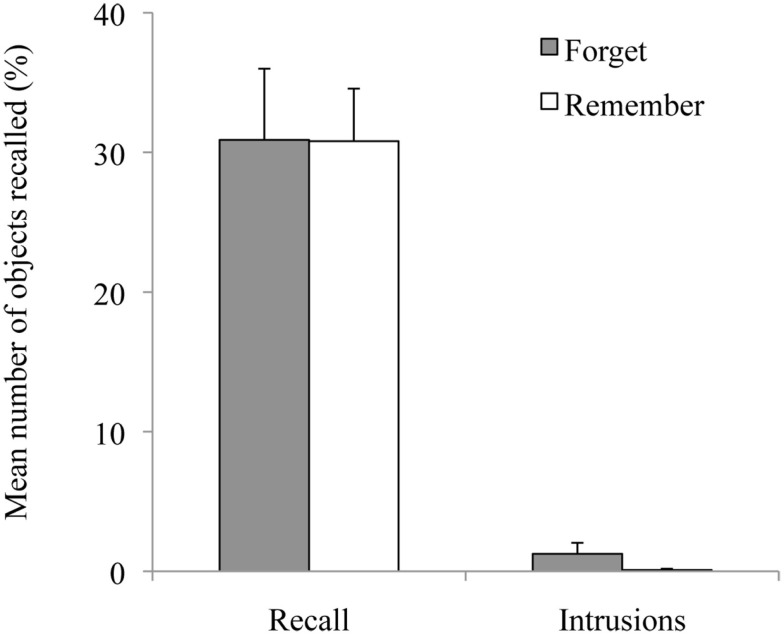
**Mean percentages of objects correctly and falsely recalled in the remember group and in the forget group in Exp. 2**. Error bars represent standard errors of means. *Note:* Participants were asked to recall objects from *Set 1*. Objects that were falsely recalled from Set 2 are labeled as Intrusions.

The number of objects recalled from Set 1 (see Figure 2) were analyzed with a mixed ANOVA with Condition as the between-subjects variable and Trial (1–4) as the within-subjects variable. There was a significant effect of Trial [*F*(3, 78) = 19.48, MSE = 0.997, *p* < 0.01], reflecting a linear increase in recalled items with trials [*F*(1, 26) = 31.02, MSE = 1.63, *p* < 0.01]. Neither the main effect for Condition nor the interaction between Trial and Condition reached significance (*F*s < 1).

#### Intrusions

As expected, intrusions were at floor levels in both groups (*M* = 1.25% for the forget, and *M* = 0.09% for the remember group) and were therefore not analyzed statistically.

### Discussion

When I asked participants to recall Set 1 right after they had encoded Set 2 in Session 2, Set 1 was recalled without intrusions in both experimental groups. This confirms that the intrusion effect takes time to develop (Hupbach et al., 2007). Most importantly, Experiment 2 allows us to distinguish between retrieval inhibition and list separation explanations for the reduced intrusions that had been observed in Experiment 1a and 1b, particularly when the forget cue was administered at the beginning of Session 2. Based on the retrieval inhibition account, it was expected that the forget cue temporarily blocked access to Set 1, and thus, recall of Set 1 should be impaired in the forget group in comparison to the remember group. Experiment 2 does not provide support for this assumption, because no group differences were found when recall was tested immediately after Set 2 encoding. One could argue that retrieval inhibition might vanish with the progression of retrieval trials. However, no difference in recall was found between the forget group and remember group for any of the retrieval trials, not even the first retrieval attempt. Thus, the reduced intrusions from Set 2 into Set 1 in Experiment 1a and 1b cannot be explained by retrieval inhibition. Alternatively, based on the contextual change account, I reasoned that recall might not be impaired in the forget group, because the internal context shifted for both groups over the 48 h delay period, and thus did not match the encoding context for either of the two groups particularly well. Although one has to be careful to interpret null effects^1^, the lack of recall differences between the forget and the remember group in Experiment 2 is in line with this reasoning. Furthermore, Experiment 2 suggests that enhanced set differentiation (reflected in reduced intrusions when tested after a delay) can unfold without impaired recall of Set 1.

As mentioned in the introduction, the DF effect has two facets, impaired memory for the to-be-forgotten material (costs), and improved memory for the material that is presented afterward (benefits). Although the two effects do not always occur together (see e.g., Bäuml, 2008), it is important to ask whether the reduced intrusion effect of Experiment 1 is accompanied by an improved memory for Set 2 objects. This was tested in Experiment 3.

## Experiment 3

The long-term benefits of DF have rarely been studied (see Liu, 2001 for an exception). The two-factor account of DF (Sahakyan and Delaney, 2005) assumes that the benefits (i.e., enhanced recall of List 2 following DF) reflect the implementation of an improved encoding strategy for List 2. Specifically, the forget instruction causes people to rethink their encoding strategies, and to choose a more sophisticated one for the new list. An improved encoding strategy should have long-term consequences (Liu, 2001). Therefore, I expected that instructing people to forget Set 1, especially immediately before Set 2 encoding, would result in improved memory for Set 2 in comparison to a control group. Intrusions from Set 1 into Set 2 recall were not expected for any of the experimental groups, because previous studies have shown that intrusions only occur in one direction (from Set 2 into Set 1, but not from Set 1 into Set 2 recall; Hupbach et al., 2007, 2009).

### Methods

The exact same methods as in Experiment 1a were used, with the exception that all participants were asked to recall Set 2 instead of Set 1 in Session 3.

### Participants

Thirty-six undergraduate students (25 females, 11 males) from Lehigh University participated in the experiment. They received course credit or financial compensation for participation. Twelve participants were randomly assigned to each condition (FC-1, FC-2, control group). The control group was not asked to remember Set 1 before learning Set 2, and thus resembles the control group of Experiment 1a.

### Results

#### Learning set 1 and set 2

A 3 (Condition) × 2 (Set) mixed ANOVA revealed a significant main effect of Set [*F*(3, 99) = 17.97, MSE = 1.20, *p* < 0.01]. Participants needed fewer learning trials to reach criterion for Set 2 (*M* = 2.22) than Set 1 (*M* = 2.61), replicating Experiment 1. Learning was comparable in all three conditions: there was no main effect of Condition, and no interaction between Set and Condition (*F* ≤ 1.38, *p* ≥ 0.27).

#### Recall of set 2

**Figure 3:**
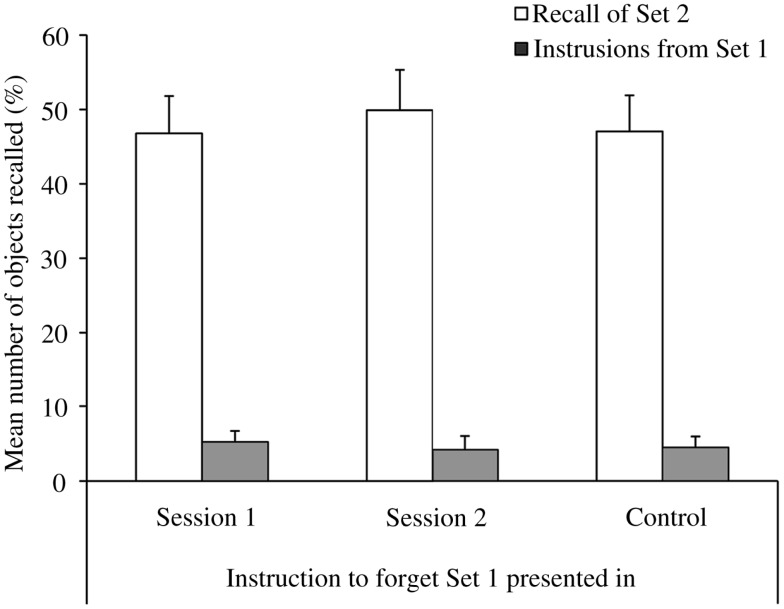
**Mean percentages of objects correctly and falsely recalled in the different experimental groups in Experiment 3: the forget cue was either given at the end of Session 1, the beginning of Session 2, or was omitted (control)**. Error bars represent standard errors of means. *Note:* Participants were asked to recall objects from *Set 2*. Objects that were falsely recalled from Set 1 are labeled as Intrusions.

The mean percentages of objects recalled from Set 2 and the mean percentages of objects falsely recalled from Set 1 (intrusions) are displayed in Figure 3. The number of objects recalled from *Set 2* was analyzed with a 3 (Condition) by 4 (Trial) mixed ANOVA. Only the main effect of Trial was significant [*F*(3, 99) = 17.97, MSE = 1.20, *p* < 0.01], showing that recall improved over the four retrieval trials [linear contrast: *F*(1, 33) = 31.45, MSE = 1.89, *p* < 0.01]. There was no effects of Condition and no interaction between Trial and Condition (both *F*s < 1).

#### Intrusions from set 1 into set 2

As expected intrusions were at floor levels in all three groups and were therefore not further statistically analyzed.

### Discussion

Experiment 3 shows that the forget cue did not improve memory for Set 2, regardless of when it was presented. This finding contradicts my hypothesis of better memory performance in the forget groups in comparison to the control group, based on the assumption that the forget instruction should have caused participants to implement a more successful encoding strategy for Set 2. However, in contrast to most DF studies, in the object-learning paradigm, participants went through several learning trials until they could recall a specified number of objects (17 out of 20 presented objects). Therefore, potential initial encoding strategy differences among the different groups might have been “washed out” over successive learning trials. Furthermore, in contrast to the costs of DF, the benefits are less reliably observed, and studies that do find List 2 enhancements exert much less control over encoding processes than in the current study, which leaves more room for individual strategy choices (see Sahakyan and Delaney, 2010 for further discussion).

Critically, and in contrast to most DF studies, the object-learning paradigm implements 48-h time delays between Set 1, and Set 2 presentation, and the final memory test. To my knowledge, Joslyn and Oakes’ (2005) study on DF of autobiographical events is the only published study that used a list-wise method and various time delays. Similar to what was found in the present study, the forget instruction did not result in enhanced remembering of the events that followed the forget instruction. Future research has to determine whether the enhanced memory for the new information is a short-lived phenomenon due to the new information being temporarily more accessible, or whether it is dependent on using less stringent encoding procedures (Sahakyan and Delaney, 2010).

Intrusions from Set 1 into Set 2 recall were not found for any of the experimental groups in Experiment 3. This replicates previous studies that have shown that intrusions only occur in one direction (from Set 2 into Set 1, but not from Set 1 into Set 2 recall; Hupbach et al., 2007, 2009). This is in line with the assumption that consolidation and reconsolidation are affected by retroactive, but not proactive interference. Alternatively, Set 2 memory was encoded more recently than Set 1 memory, and therefore, might be relatively immune to intrusions due to its strength.

## General Discussion

When memories are reactivated, they re-enter a plastic state in which they can be modified with new information. In the present study I asked whether such modifications can be prevented, that is, whether memory can be preserved, when people are instructed to forget the original memory before new information is presented. I combined the reconsolidation paradigm (that leads to integration of new information into a reactivated memory) with a list-wise DF paradigm (in which people actively attempt to forget a recently encoded memory). Experiment 1 strongly suggests that memory modifications can be prevented via forgetting instructions: the influence of the spatial context, that usually triggers the incorporation of Set 2 objects into memory for the first set (Hupbach et al., 2008; and control group of the Exp. 1a) could be overcome when people were instructed to forget the first set, especially when the forget instruction was given shortly before Set 2 encoding. This shows that DF can affect not just very recent, but also more remote and well-established memories, a finding that is new in the DF literature. The instruction to forget Set 1 might have encouraged people to focus on the differences between the two sets, which promoted increased set differentiation. Conversely, the remember instruction (or giving no specific instruction at all), might have caused people to focus on the similarities between the two episodes, promoting Set 2 integration. Indeed, Experiment 2 provides support for the view that the reduced intrusions in the forget group were due to list segregation/isolation, rather than inhibition of Set 1.

The present finding of reduced intrusions from Set 2 into Set 1 aligns with recent findings showing that the forget instruction reduces intrusions in the traditional DF paradigm in which both list are learned and retrieved within the same session (Lehman and Malmberg, 2009; Sahakyan and Delaney, 2010; but see Spillers and Unsworth, 2011). In reference to the context change account, Sahakyan and Delaney explain the reduced intrusions by assuming that the forget cue results in more distinct List 1 and List 2 contexts, which in turn “serve as better retrieval cues for items from their own list” (p. 1351). Intrusions in the present study were restricted to Set 2 intruding into Set 1, whereas especially in Sahakyan and Delaney’s study, List 1 also intruded into List 2. The finding of bidirectional intrusions might depend on presenting both lists in close temporal proximity. On the other hand, the unidirectional intrusions in the object-learning paradigm are assumed to reflect a reconsolidation process that integrates List 2 into List 1 memory. Specifically, returning to the same context or being instructed to keep remembering Set 1 in Session 2 reactivate Set 1 memory, allowing the newly presented Set 2 items to become incorporated into Set 1 memory (Hupbach et al., 2007, 2008).

Interestingly, instructing people to forget Set 1 in Session 2 has similar effects as a spatial context change between List 1 and List 2 presentation. Hupbach et al. (2008) found intrusions from Set 2 into Set 1 memory only when Set 1 and Set 2 were encoded in the same spatial context, but not when Set 2 was encoded in a different room. In the present study, spatial context was held constant across all three conditions. However, intrusions were prevented by the forget cue. This points to an interesting interplay between internal and external context in the object-learning paradigm. It seems that the internal context change can counteract the influence of the spatial context. This is especially interesting in the light of the failure to accomplish the opposite, i.e., to mentally reinstate the spatial context in a different environment: when placed in a different spatial context in Session 2, mentally reinstating the encoding context of Set 1 did not lead to intrusions from Set 2 into Set 1 in Session 3 (Hupbach et al., 2008).

The study raises the general question of boundary conditions for memory updating and reconsolidation. It has been proposed and experimentally demonstrated that reconsolidation is only triggered when there is a mismatch between what is expected based on prior learning and what happens after the reminder (prediction error; Morris et al., 2006; Lee, 2009; Sevenster et al., 2012). Another boundary condition for memory reconsolidation is the strength of a memory. Animal studies have shown that strong fear memories, when reactivated, are initially resistant to reconsolidation (Suzuki et al., 2004; Wang et al., 2009). In a recent study, Hupbach et al. (2010) “overtrained” participants on Set 1 by increasing the number of encoding trials. Overtraining markedly reduced intrusions. Thus, stronger memories are somewhat resistant to change, although not completely impermeable. And yet in other situations, updating or integration requires verbal instructions. In a paired-associates learning paradigm, the integration of new paired-associates into the reactivated memory crucially depends on giving participants a verbal instruction to do so. Without the verbal instruction to add the new information, the second list interfered with memory for the first list (Forcato et al., 2010).

Taken together, the present study demonstrates for the first time that deliberate attempts to forget a memory can immunize it such that the presentation of potentially interfering material has no effect, and the memory can be recalled unchanged in the future. This way, the attempt to forget can have beneficial effects on memory. This finding may become quite relevant in the context of eyewitness testimony, where long-term memory modifications can be troublesome and highly consequential (e.g., Loftus, 2005). Chan et al. (2009) have shown that recalling a witnessed event before presenting misleading information enhances the misinformation effect, that is people are more likely to adopt false details as their true memories in a later memory test. The present study suggests that the instruction to forget the witnessed event before presenting related information might protect the memory from being altered.

## Conflict of Interest Statement

The author declares that the research was conducted in the absence of any commercial or financial relationships that could be construed as a potential conflict of interest.

## Footnotes

^1^The observed difference between the forget- and remember-group was extremely small (0.7 items, or 3.5%). A *post hoc* power analysis showed that given the group sample sizes (*N* = 14) and observed SD (2.77 and 2.78), the paired sample *t*-test had a power of 0.72 to detect a difference of 12% (a rather conservative estimate, based on a recent study by Pastötter and Bäuml, 2010). Thus, the lack of statistical significance is unlikely due to a lack of power.
